# Structure preserving adversarial generation of labeled training samples for single-cell segmentation

**DOI:** 10.1016/j.crmeth.2023.100592

**Published:** 2023-09-18

**Authors:** Ervin Tasnadi, Alex Sliz-Nagy, Peter Horvath

**Affiliations:** 1 Synthetic and Systems Biology Unit, Biological Research Centre, Eötvös Loránd Research Network, 6726 Szeged, Hungary; 2 Doctoral School of Computer Science, University of Szeged, 6720 Szeged, Hungary; 3 Single-Cell Technologies, Ltd, 6726 Szeged, Hungary; 4 Institute for Molecular Medicine Finland (FIMM), University of Helsinki, 00014 Helsinki, Finland

**Keywords:** data augmentation, single-cell segmentation

## Abstract

We introduce a generative data augmentation strategy to improve the accuracy of instance segmentation of microscopy data for complex tissue structures. Our pipeline uses regular and conditional generative adversarial networks (GANs) for image-to-image translation to construct synthetic microscopy images along with their corresponding masks to simulate the distribution and shape of the objects and their appearance. The synthetic samples are then used for training an instance segmentation network (for example, StarDist or Cellpose). We show on two single-cell-resolution tissue datasets that our method improves the accuracy of downstream instance segmentation tasks compared with traditional training strategies using either the raw data or basic augmentations. We also compare the quality of the object masks with those generated by a traditional cell population simulation method, finding that our synthesized masks are closer to the ground truth considering Fréchet inception distances.

## Highlights

• We propose a deep-learning method for simulating cellular imaging data
• Our pipeline uses StyleGAN2-ada to learn instance masks directly from data
• This method is suited to the complex object layouts of biological organs and tissues
• The synthesized samples can be used to augment the initial training dataset

## Motivation

Generative construction of novel training samples has been shown to improve the test accuracy of segmentation networks in the medical image analysis domain. However, only one or a few objects (e.g., organs, malignant tumors) are segmented in medical images, and thus semantic segmentation is typically used. In contrast, in bioimage analysis, several objects (e.g., single cells) should be retrieved, and thus instance segmentation methods are employed. We sought to adapt the previously proposed methods for generative augmentation in medical image analysis to bioimage analysis by learning discrete object masks and the corresponding microscopy images directly from the data itself.

## Graphical abstract

**Figure:**
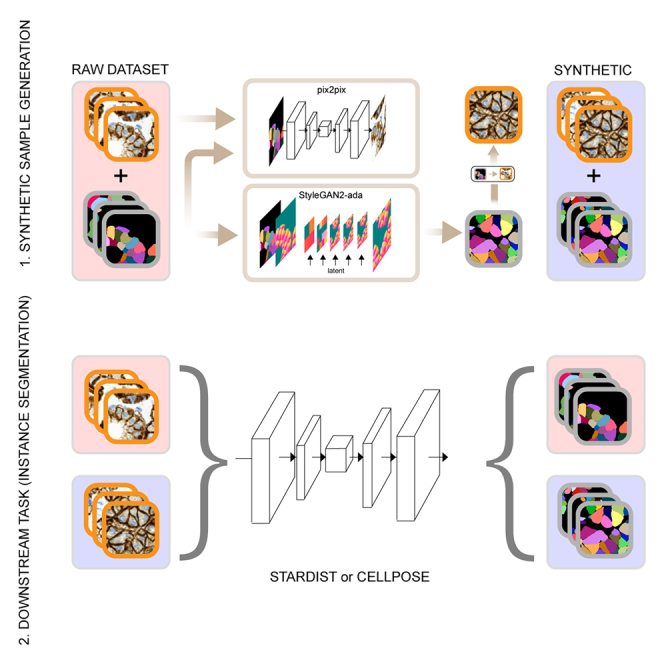

## Teaser

Tasnadi et al. propose a generative approach to model the joint distribution of instance masks and the corresponding microscopy images of a training dataset. The method can be used for simulating tissue microscopy images and masks of cell populations or automatic augmentation when combined with the initial training set.

## Introduction

Data augmentation is one of the simplest ways to improve the generalization capability of convolutional neural networks. The motivation behind data augmentation is the fact that, using the appropriate transformations, one can generate artificial elements from the original dataset that can improve the generalization capability of the model when jointly used with the raw training data. Data augmentation is particularly relevant for biological and medical image analysis, where primary data may be limited or costly to obtain. Common transformations used for data augmentation in biological image analysis are simple affine transformations like rotation, translation, scaling, and nonlinear transformations, for example, elastic deformations that equally affect both the input and target images. Other transformations like the addition of (Gaussian) noise or intensity transformations affect the input image only and leave the segmentation untouched.

To simulate microscopy images and masks of cell populations, several methods have been proposed. The SIMCEP^1^ method aims to generate realistic-looking cell populations in two steps. In the first step, the vertices of a regular polygon are perturbed by normally distributed displacements. Then, a cubic spline is fitted to the vertices of the transformed polygon. The instances are then placed on an empty canvas. Each object is assigned to a cluster with uniform probability, while the in-cluster object-centroid distances are distributed normally and the location of the centroids are uniformly distributed in the image. Another related tool is the Cytopacq^2^^,^^3^ method, which aims to simulate the whole imaging pipeline. The approach is also capable of generating 2D/3D digital “phantoms” of HL-60 cells (among others) by deforming a sphere or ellipsoid using fast level set methods with random noise. Although these approaches can model simple cell populations, the positions of the objects are still drawn from simple parametric distributions, and thus they cannot capture more complex layouts.

Another class of methods utilize generative adversarial networks (GANs) for automatic data augmentation. Many of the related methods aim to solve segmentation or classification tasks for medical images^4^^,^^5^^,^^6^^,^^7^^,^^8^^,^^9^^,^^10^ or biological images.^11^^,^^12^^,^^13^^,^^14^ Methods developed for medical image classification learn a model for each class^8^^,^^10^^,^^15^ and then draw examples from the learned distribution to solve class imbalance problems. It has been demonstrated that a single model is also effective if conditioned on the class labels.^9^ Unpaired image-to-image translation can be also exploited to bridge the domain shift (contrast and noncontrast images) between the training and test sets.^7^ Other methods developed for segmentation of medical images learn the joint distribution of the actual image and the corresponding segmentation. The simplest way is to train a GAN model on the initial training set and then draw samples from the learnt distribution.^4^^,^^6^ A semi-supervised approach extends this model by predicting geometric and intensity transformations to be applied on the training set to synthesize elements more similar to the distribution of the test set.^5^ Learning the joint distribution of the images and masks is shown to be effective in medical images, where usually one or a few target objects are segmented. In contrast, in biological images, usually many distinct objects should be simulated, and the goal is to reproduce discrete instance masks. Nevertheless, the naive approach is proposed for synthesizing binary nuclei masks for microscopy images.^13^ Other methods, however, synthesize instance masks using simple parametric methods and then use an image-to-image translation model to generate the corresponding microscopy images. An unpaired image-to-image translation model can be trained directly on the synthesized masks and the microscopy images found in the test set.^12^ Another method trains a paired image-to-image translation model using weak segmentations generated to the test.^11^ If the instance masks are not required, then a style transfer model can be directly applied on the feature level.^14^ The main limitation of the latter group of methods is that these are not easily applicable to datasets where global tissue structures result in complex object layouts since the masks are simulated using simple parametric methods. We therefore sought to design a method that learns instance masks from the data directly and that can synthesize masks where the layout of the objects has a unique structure that cannot be captured with previous methods using parametric cell population simulation.

We therefore developed a GAN to learn instance masks directly from the training data. An image-to-image translation task is then solved to transform the synthesized masks into the corresponding microscopy images. We show that in order to learn discrete masks directly from the training data with convolutional neural networks, employing a proper encoding technique is essential, even in simpler images like object masks of nuclei instances in cell cultures. The resulting synthetic samples can be combined with the starting dataset and used to train an instance segmentation network (Figure 2). Because our method explicitly returns instance masks, it can also be a drop-in replacement for traditional cell population simulation methods. It offers increased expressive power, as it can capture distributions that cannot be otherwise captured using simple parametric methods.

**Figure 1:**
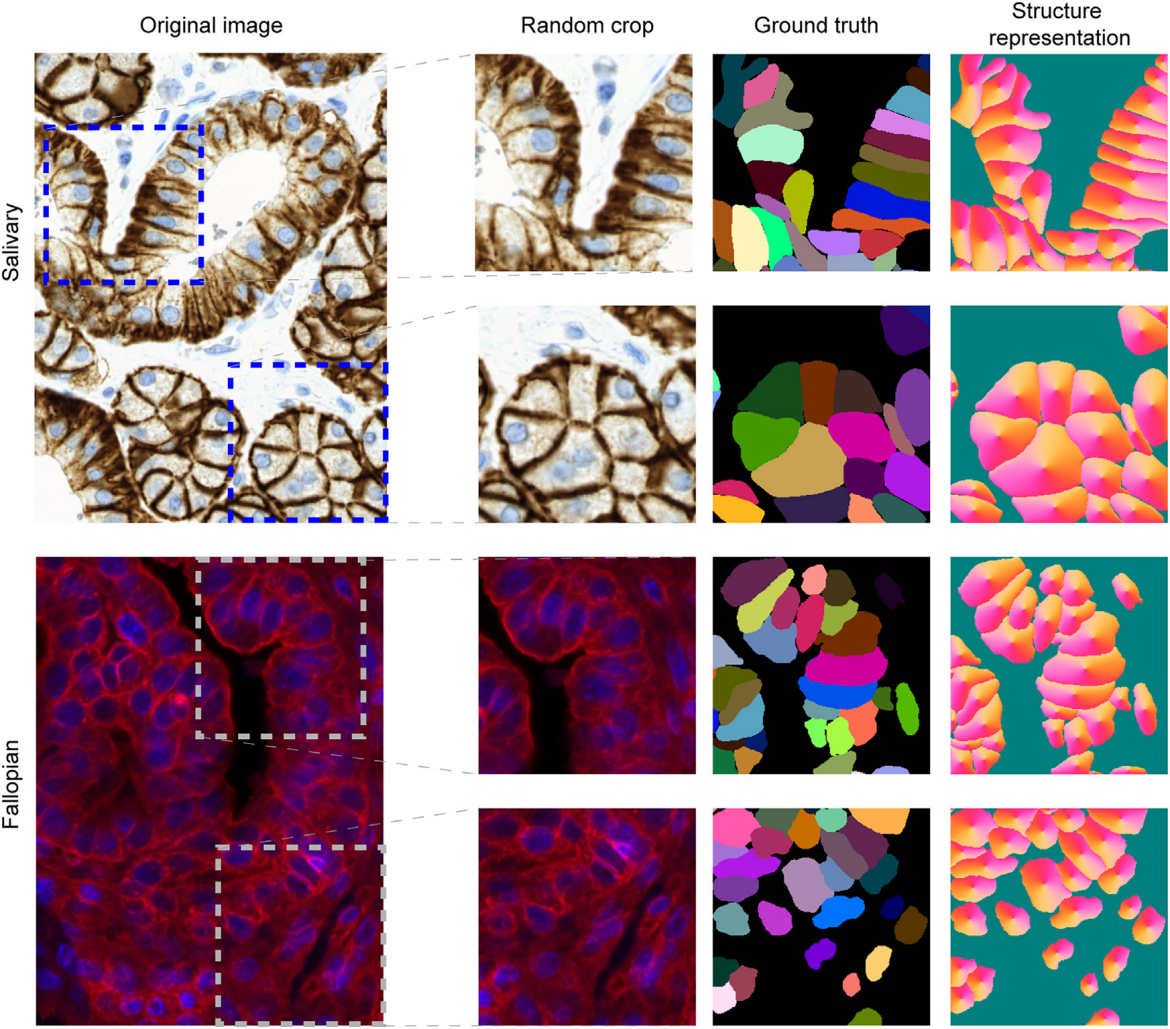
Datasets Top: salivary gland; bottom: fallopian. The datasets are from Mund et al.^21^ The right side shows the input image crops, the corresponding labeled masks, and the structure representation of the masks first proposed in the Cellpose article.^17^

We use two single-cell datasets (Figure 1) to design and validate our method. The first is extracted from a salivary gland tumor sample, and the second is a fallopian tube biopsy. The cell boundaries are annotated by a field expert. Both datasets have a particular global structure considering the layout of the cells. We found that these complex global structures cannot be easily captured using previous methods, while our convolutional neural network-based approach can deal with these types of data.

## Results

**Figure 2:**
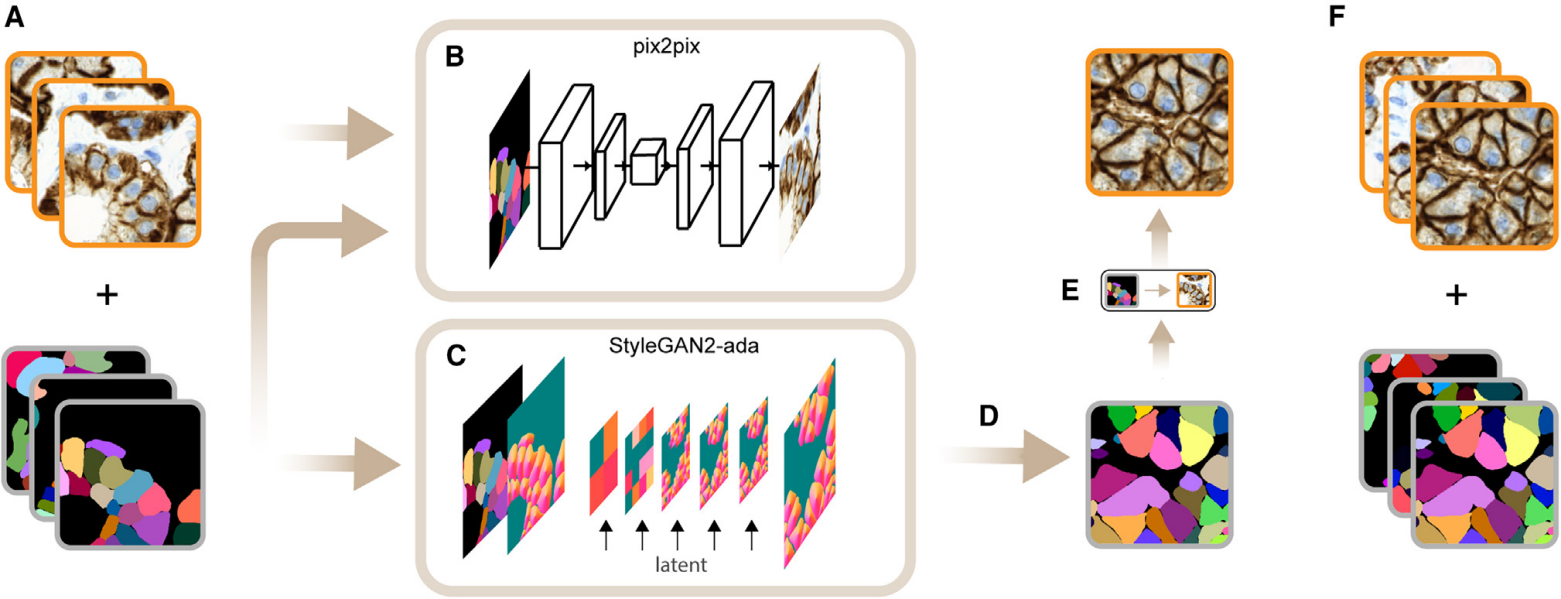
The proposed model (A) The crops from the original dataset with the input microscopy images and their corresponding ground-truth masks that are the result of EXTRACT-CROPS(S). (B) Learning an image-to-image translation model, $m_{pix2pix}$, to translate the masks into the microscopy images in the training set: the result of the function call PIX2PIX-TRAIN($\left\{m_{j}^{c}\right\}$, $\left\{i_{j}^{c}\right\}$). (C) A StyleGAN2-ada model, $m_{StyleGAN2-ada}$, is trained on the heat-flow representations of the masks in the training set: the result of STYLEGAN2-ADA-TRAIN(ENCODE ($\left\{m_{j}^{c}\right\}$)). (D) The learned StyleGAN2-ada model is then used to generate heat flows from the distribution learned from the training set, which are then converted back into labeled masks: DECODE(STYLEGAN2-ADA-GENERATE($m_{StyleGAN2-ada}$, K). (E) The learned style transform model (the result of B) is then applied on the synthetic masks by calling PIX2PIX-GENERATE($m_{pix2pix}$, $\left\{m_{k}^{s}\right\}$). (F) The synthetic dataset with the synthetic masks and the corresponding microscopy images (noncurated images). See Pseudocode 1 for the details.

Our approach uses a GAN and image-to-image translation to synthesize novel samples that mimic the original dataset; therefore, it can be considered a fully data-driven approach. First, a state-of-the-art GAN is used (StyleGAN2-ada^16^ in our case) to learn the distribution and shape of the instances in the mask images of the training set after applying a general encoding technique to encode the masks in order to be able to be successfully learnt by the network. We show that an encoding technique is crucial when learning the masks because of their discrete nature. For the encoding, we use the Cellpose’s heat-flow encoding.^17^ Then, synthetic heat flows are generated using the learnt GAN model, which are decoded in a subsequent step to discrete masks. In parallel to the GAN training, an image-to-image translation task is solved using the pix2pix^18^ method to learn the translation from the masks to the microscopy images in the training dataset. Then, the learnt pix2pix models are used to construct the corresponding synthetic microscopy image for each synthesized mask. The resulting synthetic samples can be combined with the starting dataset and used to train a segmentation network (Figure 2). Our method, however, can not only be used for data augmentation but can replace traditional cell population methods when only the masks are needed (Figure 2; Pseudocode 1).

### The proposed model for synthetic sample generation

Our method implements the GENERATE-SYNTHETIC-SAMPLES procedure (Pseudocode 1). It operates on the training dataset $S={\left(\left\{i_{i}\right\},\left\{m_{i}\right\}\right)}_{i=1}^{N}$, where $\left\{i_{i}\right\}$ is the microscopy images and $\left\{m_{i}\right\}$ is the corresponding segmentation masks. Each pixel in the segmentation mask encodes the instance with the intensity, while the intensity 0 encodes the background.

**procedure** GENERATE-SYNTHETIC-SAMPLES(S, K)

  ${\left(\left\{i_{j}^{c}\right\},\left\{m_{j}^{c}\right\}\right)}_{j=1}^{M}$ = EXTRACT-CROPS(S)

  $m_{StyleGAN2-ada}$ = STYLEGAN2-ADA-TRAIN(ENCODE ($\left\{m_{j}^{c}\right\}$))

  ${\left\{m_{k}^{s}\right\}}_{k=1}^{K}$= DECODE(STYLEGAN2-ADA-GENERATE($m_{StyleGAN2-ada}$, K)

  $m_{pix2pix}$ = PIX2PIX-TRAIN($\left\{m_{j}^{c}\right\}$, $\left\{i_{j}^{c}\right\}$)

  $\left\{i_{k}^{s}\right\}$ = PIX2PIX-GENERATE($m_{pix2pix}$, $\left\{m_{k}^{s}\right\}$)

  **return**$\left\{i_{k}^{s}\right\}$,$\left\{m_{k}^{s}\right\}$

**end procedure**

Pseudocode 1: the pseudocode of our method. It crops M overlapping patches from the input samples (M is determined automatically) and then synthesizes and returns K samples that are assumed to be similar to the crops extracted from the N input images. The ENCODE function converts the labeled masks into heat-flow representation, and the STYLEGAN2-ADA-TRAIN function represents the training on the heat flows resulting in a GAN model, $m_{StyleGAN2-ada}$. The resulting GAN model is used to synthesize flows using STYLEGAN2-ADA-GENERATE, which will be converted back to labeled masks using DECODE. The function PIX2PIX-TRAIN learns the transformation $\left\{m_{j}^{c}\right\}\to \left\{i_{j}^{c}\right\}$, resulting in an image-to-image translation model, $m_{pix2pix}$, that will be used to synthesize the corresponding microscopy images to the already synthesized masks using PIX2PIX-GENERATE. In the upper indices, s stands for the synthesized crop image, and c is a crop from the starting dataset.

We first extract overlapping crops of size 256×256 from the input images and masks using the EXTRACT-CROPS function (Pseudocode 1). A crop (with the corresponding image) is kept if it contains at least a few distinct objects; otherwise, it is discarded (K is the maximum number of crops successfully extracted). We also add the orthogonally rotated transformations of each crop to the dataset.

To generate the synthetic masks, we learn the distribution of the instances in the original dataset and then use the learned model to generate objects from the distribution. Previous works generate the synthetic masks by sampling objects from a cell database and then placing them on an empty canvas after applying random transformations on them (rotation, resize, etc.).^11^^,^^12^ However, this type of approach may not be able to model complex distributions where the instances follow unique global tissue structures as in our case (Figure 1). This is often the case in tissue samples. Therefore, we train a GAN on the masks to model not only the shape of the objects but their relative locations and orientations. As our method is intended to work on small, annotated datasets, we have to train a GAN with a limited number of samples. Fortunately, recent GANs offer nonleaking data augmentation to learn from limited-size datasets.^16^ In our proposed pipeline, we use the StyleGAN2-ada framework for all the experiments: first, a mask model, $m_{StyleGAN2-ada}$, is learned using the STYLEGAN2-ADA-TRAIN function, and then the synthetic masks are drawn using the learned model.

**Figure 3:**
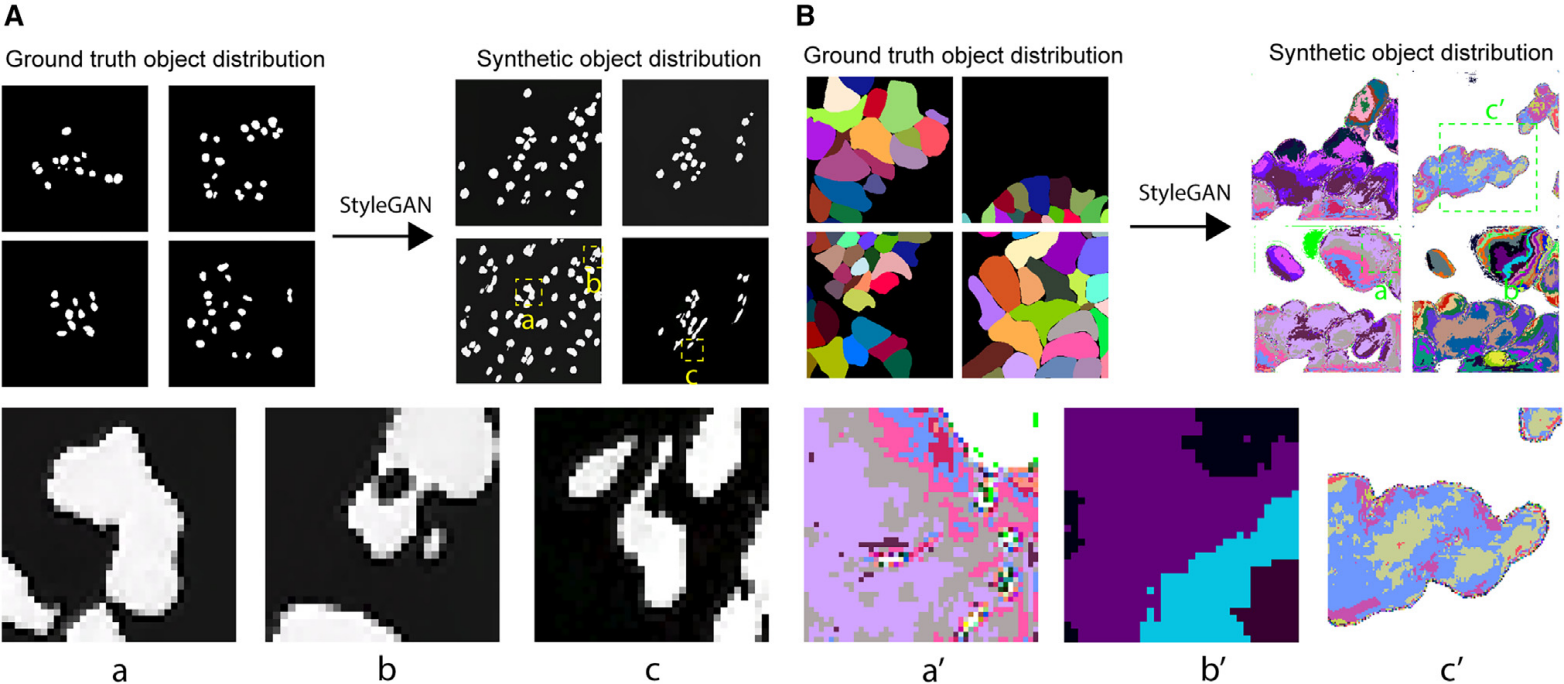
Common errors when training StyleGAN2-ada directly on the masks Top: (A) the StyleGAN2-ada was trained on the binarized masks from the DSB2018^20^ dataset (intensity 0 marks the background, while 255 marks the foreground). (B) Trained on the labels of the salivary gland dataset directly. We did not do any preprocessing on the labeled masks; we just trained the network on them. Bottom: common issues when training directly on the binarized masks: (a) the objects cannot be separated; the StyleGAN2-ada generates blobs instead of instances, (b) holes between the objects, and (c) fragmented objects with blurry boundaries. When training on the labels, the common issues are (a′) holes in the objects, (b′) nonuniform intensities represent an object, and therefore the reconstruction is nearly impossible, and (c′) nuclei blobs, containing mixed intensities (the input is grayscale in the labeled case, and the colors are only added for better visualization).

To synthesize the masks, a naive solution would be to feed the binarized version of the instance masks ($\left\{m_{j}^{c}\right\}$) directly into the GAN, where all of the cells share the same label,^13^ while the background is marked with zeros (that is, the ENCODE function in Pseudocode 1 is the threshold function with an appropriate threshold parameter). This strategy is suitable for tasks where only one or a few objects should be segmented, but in instance segmentation, especially in single-cell segmentation of tissues where objects densely located, this strategy has two main limitations: (1) instances that share boundaries with other instances are indistinguishable from each other, and therefore touching cells cannot be modeled. (2) We observed that if a model is trained on the binarized masks, the network generates lots of fragmented objects, similarly to the presented graphical results in the referenced paper, where the authors also used binary mask representation.^11^ We also observed this error, and this particular failure mode is presented in Figure 3A.

We also experimented by feeding the GAN with raw labeled masks where each object is encoded with a unique intensity value (the ENCODE function is identity). Although, this way, the generated cell instances are not fragmented, small intensity variance can be observed in almost each generated object that is nearly impossible to fix, and consequently, touching objects cannot be modeled. This happens because the input has an inherently discrete property that is not respected by the StyleGAN2-ada, as it learns the proper (continuous) distribution of pixels that results in perceptually appealing results, but no terms in the loss forces the dynamics of the learning to respect the discrete nature of the dataset. Results and common failure modes of these naive approaches are presented in Figure 3.

To overcome the issues above, we choose to encode our labeled masks into a dense and continuous structure representation to solve both problems. In theory, many representations could work (see the note below), but we found Cellpose’s^17^ heat-flow simulation to be the most robust for our task: in the encoding process, the centroid of each cell is determined, and a constant heat is applied to that point in an iterative manner (Figure 1, rightmost column). The heat distribution is captured at the end of this iterative process, and the objects are represented using the gradients of the final heat distribution. Reconstruction (decoding) is done by following the gradients for each pixel: if two different pixels converge to the same position, then they are representing the same object. We convert each mask into their corresponding vector-flow representation and feed them to the StyleGAN2-ada during training. The converted masks are encoded in three channels: two channels represent the gradient of the flow (dx and dy) in each pixel, and the third channel encodes the object probability. The main advantage of this representation is that the instance masks can be represented as 3-channel images, and there is no need for architectural changes in the StyleGAN2-ada to feed the masks in the vector-flow format. The vector-flow representation naturally solves problem 1 since the pixels near the touching region converge to the reference points (centroids) of the objects they are part of. Our experiments show that StyleGAN2-ada can learn the vector flows and that the synthesized images can be decoded by the simple algorithm above. Based on our experiments, problem 2 is also solved, as we did not observe the fragmented objects in the generated vector flows (Figure 3). After training the GAN with the flows, we generate synthetic flows and decode them with the mentioned tracking algorithm, and thus we get synthesized discrete masks.

Our method does not explicitly depend on the Cellpose representation. In theory, any representation may work that can encode a labeled mask into a dense image. We also experimented with gradient vector-flow representation^19^ but found that the Cellpose representation has higher tolerance on the inaccuracies generated by the GAN.

Parallel to training the StyleGAN2-ada model, we learn $m_{\text{pix}2\text{pix}}$ using image-to-image translation on the training set^18^ that will be later used to transform the synthesized masks into their corresponding synthetic microscopy images. One can learn the mapping of the vector-flow representation of the microscopy images directly (the first parameter of PIX2PIX-TRAIN is the raw output of STYLEGAN2-ADA-GENERATE), but we observed that learning the translation from the raw labeled masks into their corresponding microscopy images leads to better image quality in the datasets we are working on. After both the image-to-image translation task and the synthetic mask generation task are completed, the pix2pix model is used to translate the synthetic masks into the corresponding synthetic microscopy images, and both sets are returned. The whole pipeline is shown in Figure 2 and summarized in Pseudocode 1.

The synthesized images and masks returned by the method can be used to augment the initial training set. We use the StarDist and Cellpose instance segmentation methods to demonstrate that the synthesized samples can improve the generalization capability of these networks. Although we tested the effectiveness of our augmentation policy with the networks above, our method does not depend on any particular instance segmentation method.

### Performance evaluation

We first qualitatively show that training on the binarized masks or the labels lead to poor mask quality. We trained StyleGAN2-ada on binary and labeled masks (the ENCODE function is the thresholding or identity, respectively). The binary training is tested on the masks of the DSB 2018 dataset,^20^ where only a few instances touch each other per image. We also tested the quality of the synthetic masks when the network was trained on the raw labeled images of the salivary gland dataset. In the latter, the instances follow a denser layout (compared to the DSB 2018 containing mainly cell cultures), and most of the instances share boundaries with others. Figure 3 presents the most common failure modes when training the network with these strategies.

**Figure 4:**
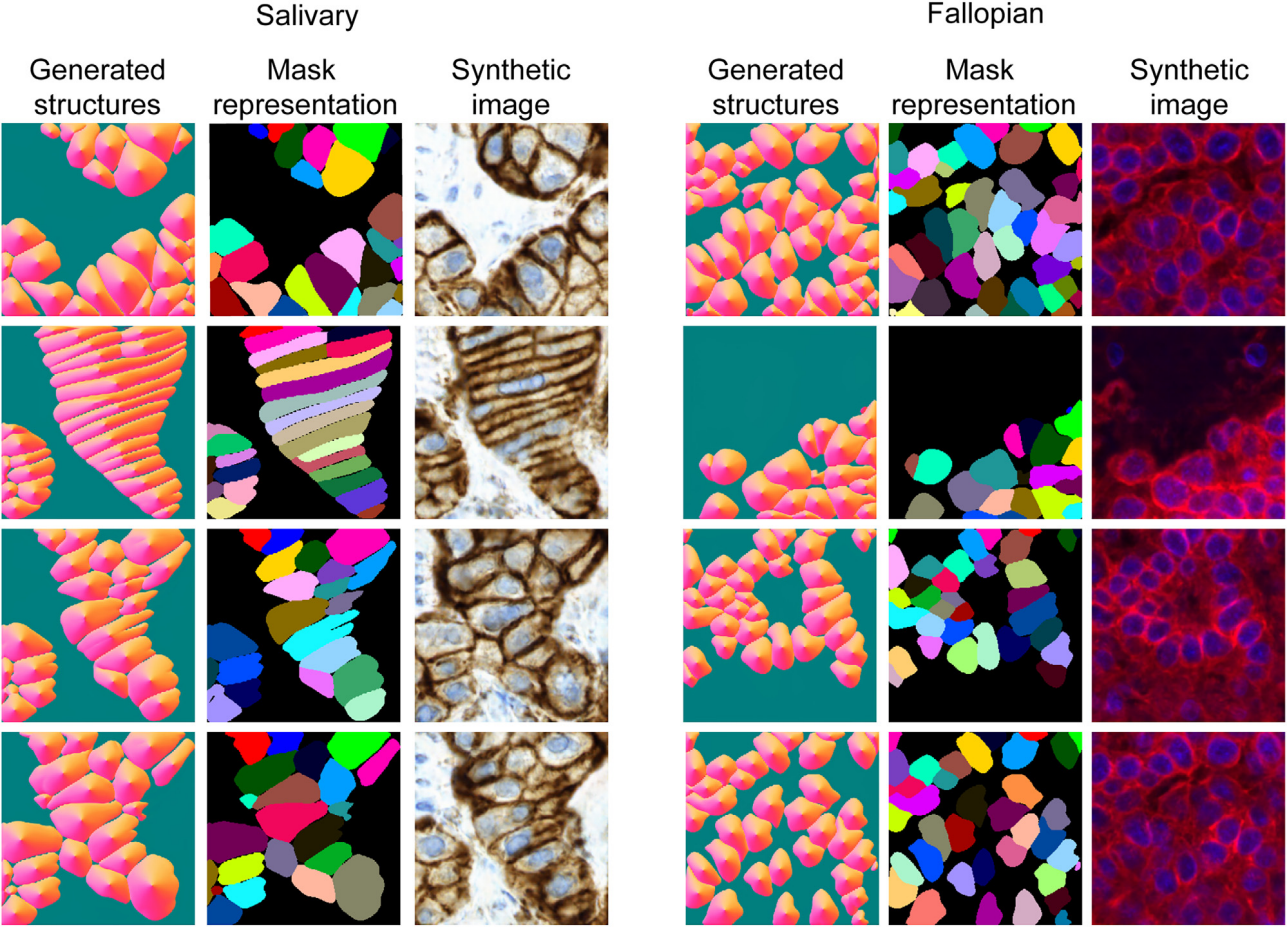
Synthesized flows, the reconstructed masks, and their corresponding microscopy images generated by our method

**Figure 5:**
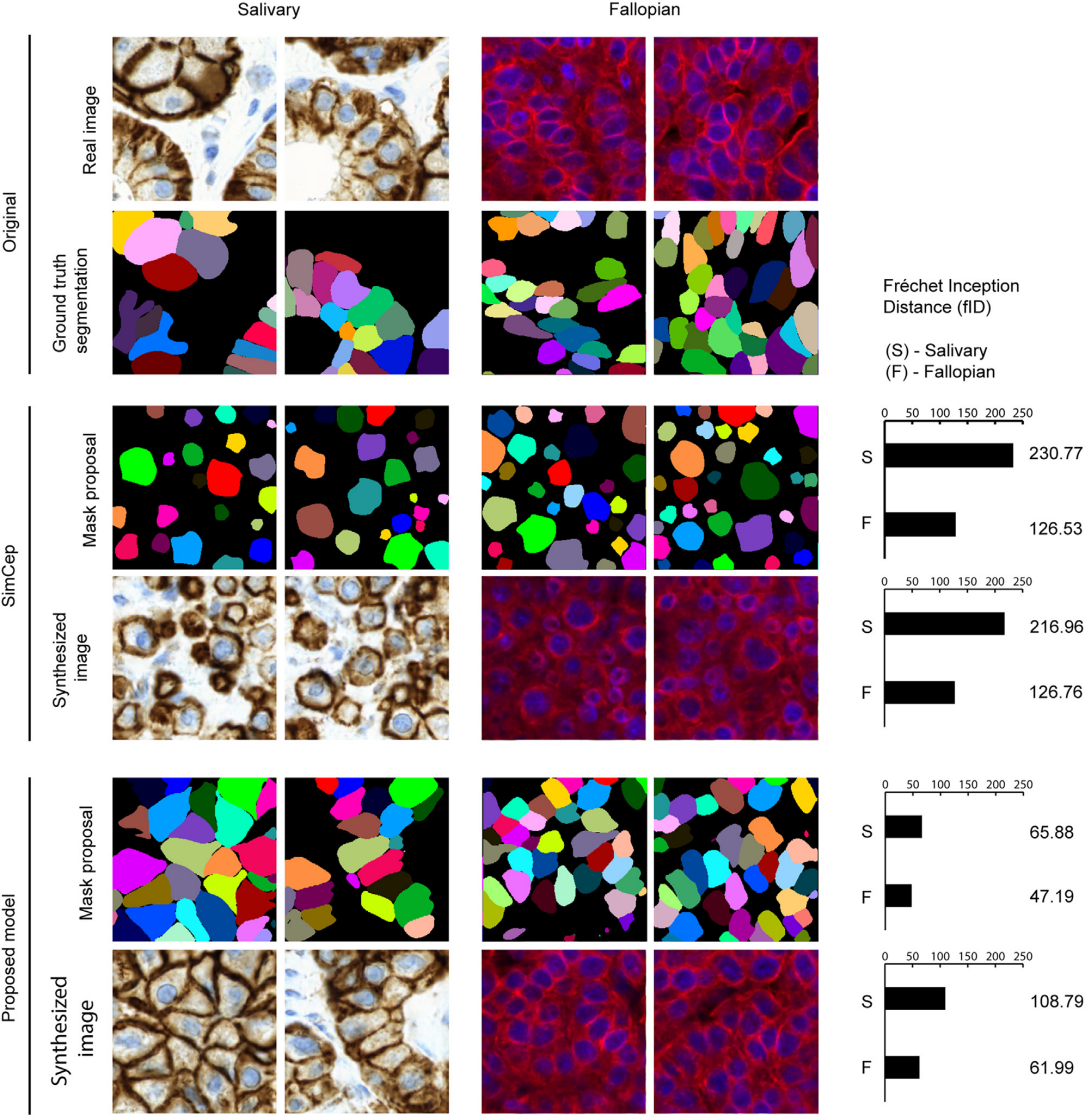
Quantitative evaluation of the synthesized masks and their corresponding microscopy images generated by SIMCEP and the proposed model The plot in the right column shows the Fréchet inception distance (fID) between the synthetic mask (microscopy image) and ground-truth mask (microscopy image). From top to bottom: distance between ground-truth mask and SIMCEP synthesized mask; distance between ground-truth microscopy image and simulated microscopy image using pix2pix with SIMCEP synthesized mask input; distance between ground-truth mask and StyleGAN2-ada synthesized masks; and distance between ground-truth microscopy image and simulated microscopy image using pix2pix with StyleGAN2-ada synthesized mask input.

Next, we compare the masks generated using the SIMCEP method with those generated with our GAN-based generation strategy by first transforming each dataset (generated by SIMCEP and ours) to the structure representation and then compute the Fréchet inception distance (fID) of the generated flows to the ground-truth flows. We adjusted each possible hyperparameter of the SIMCEP to the parameters of the ground-truth dataset (mean number of cells in each mask, min/max cell radius, estimated number of clusters) and generated a dataset of similar size to the ground truth. Based on the results, we observe that the SIMCEP generator can achieve substantially better scores if the masks have simple structure, but it still fails to generate complex structures. This is obvious from the fIDs reached by the method and by visually inspecting the generated masks. When using the GAN-based mask generation approach, the increase in the fID score is 3-fold on the salivary gland dataset, and it is still 2× when comparing the fallopian tube masks to the ground truth (Figures 4 and 5). This is not surprising, as the salivary gland dataset has a richer global structure (Figure 1).

We also compare the quality of the generated microscopy images when the learned style models are applied to the masks generated by SIMCEP and to the masks generated by our GAN-based method. Again, the fID score is substantially better on the fallopian tube dataset when using SIMCEP, as the cell structures found in this dataset are less complex. On the other hand, the fID scores are much worse when comparing the generated images from the salivary gland dataset to its ground truth. When using the masks generated by our method, a 2-fold increase can be observed in the fID scores for both datasets (Figures 4 and 5).

Our quantitative and qualitative results (Figures 4 and 5) show that our approach is useful when the layout of the objects follows a complex distribution, and it cannot be easily approximated using the SIMCEP method. In contrast, the StyleGAN2-ada generator implicitly learns the distribution of the objects in the training set that can be used later to generate more realistic microscopy images compared with what can be achieved by utilizing the masks generated using the SIMCEP method.

To show that the synthesized samples can be used to improve the instance segmentation accuracy, we trained StarDist and Cellpose models on both datasets. Two experiments were performed. In the first one, we pretrained the instance segmentation network on the synthesized images only, and then the network was fine-tuned on the raw dataset. In the second experiment, we simply merged the raw dataset and the synthesized samples. We executed the pix2pix and instance segmentation network trainings with different training-set sizes to test the effectiveness of our method on an even more limited number of samples (subset experiment). In both experiments, we cross-validated our results. We formed 5 folds on the salivary gland dataset and 4 folds on the fallopian tube.

We trained StarDist and Cellpose models on both datasets. Table 1 shows the instance segmentation results using the DSB 2018 metric^20^ (see the supporting table in the relevant section in the STAR Methods) when we first trained the networks on the synthesized images and then fine-tuned on the raw dataset.

**Table 1:** Segmentation results with StarDist and Cellpose

	Salivary gland		Fallopian tube	
	StarDist	Cellpose	StarDist	Cellpose
Raw training set	0.3443	0.4867	0.2484	0.3822
Augmentation	0.3854	–	0.3310	–
Fine-tune + augmentation	0.3893	0.4876	0.3567	0.3864

**Figure 6:**
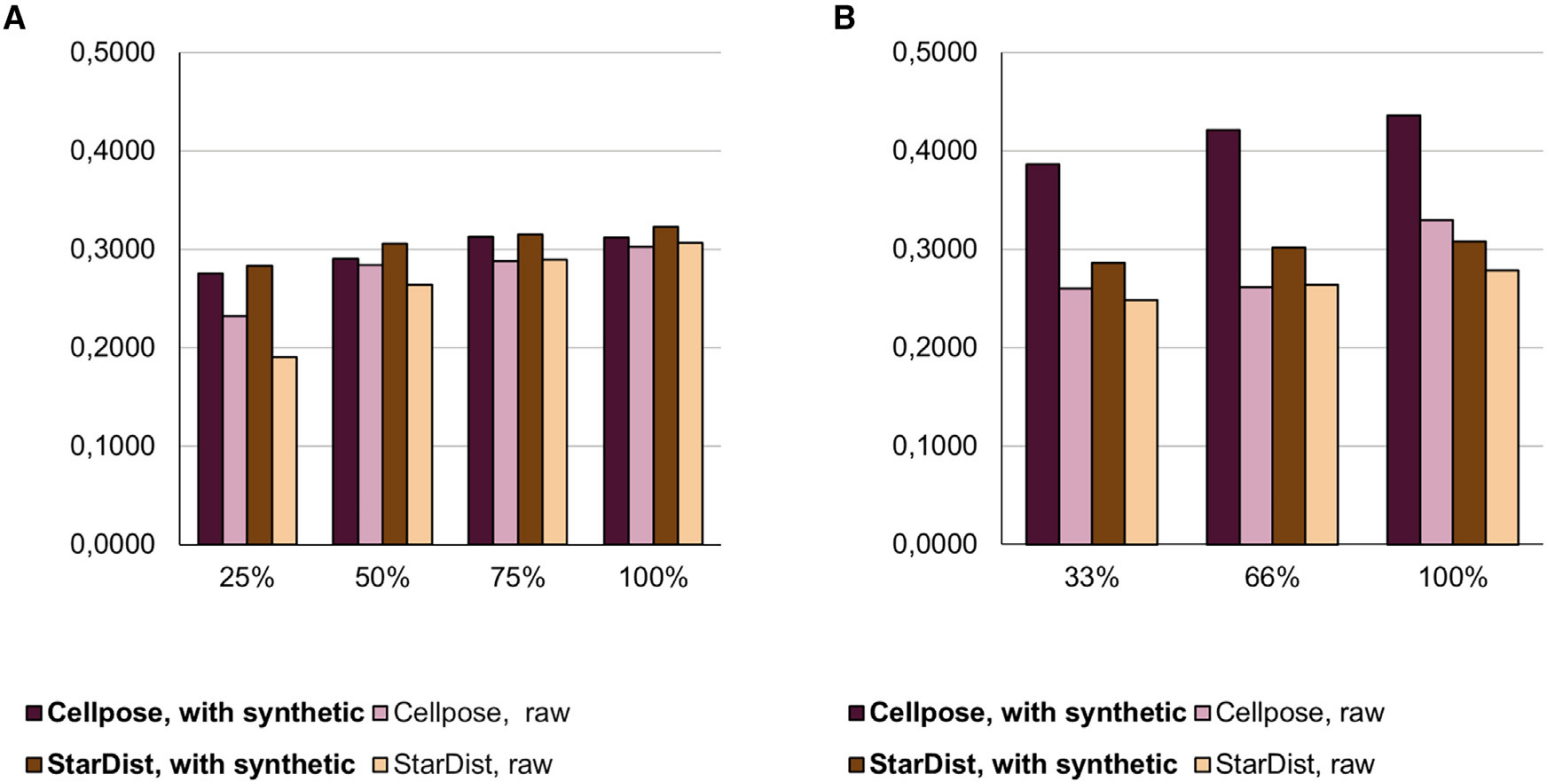
Subset experiment results (A) Fallopian tube. (B) Salivary gland. The numbers are the mean of the accuracies computed on each fold. Our augmentation protocol improves the accuracy on each subset and each fold.

For testing the accuracy on the subsets, we formed another 5 and 4 folds from the datasets and progressively eliminated images from the training set and synthesized the samples with pix2pix using only the reduced datasets. For the salivary gland dataset, we used training sets with 8 images (100% of the annotated images), 5 images (62,5%) and 3 images (37,5%). For the fallopian tube dataset, we considered 9 images as 100% and used subsets of sizes 3 and 6. The segmentation task was then executed on datasets where the synthetic images were merged to the original training set. In all the experiments, the test accuracy was higher when the segmentation network was trained on the combined dataset (Figure 6). We also observed that the standard deviation of the accuracies of the repeated experiments on each fold was substantially lower when the synthetic samples were used (see the deposited table in the shared repository in the key resources table; see Figure 6 and Table S5).

## Discussion

In this article, we introduced a synthetic sample generation strategy for instance segmentation that consists of the generation of synthetic masks using a GAN (StyleGAN2-ada) and their corresponding synthetic microscopy images using image-to-image translation (pix2pix). Our method generates the labeled masks explicitly, and they can be used for other tasks as well. We showed that the distribution of the instances on the masks generated by the GAN are more similar to the ground-truth distribution compared with the masks generated by classical parametric methods like the SIMCEP, and the GAN generation is especially useful when the underlying global structure of the objects are more complex than cell cultures like in our case. We qualitatively showed that the naive training of GANs on binary masks or the raw labels lead to suboptimal results, as fragmented objects are often produced even in images from simple cell cultures and the reconstruction of the labels is almost impossible from the GAN output, but appropriate dense mask encoding overcomes these issues. We also showed that the generated samples can be used to improve the accuracy of the downstream instance segmentation task, especially when only a very limited number of samples are available, compared with the case when only the raw dataset is used for training .

### Limitations of the study

Our proposed method solves two problems. First, it extends GAN-based augmentation approaches developed for medical image segmentation to instance segmentation, thus making it possible to apply the idea of automatic augmentation for single-cell segmentation. We tested our method on microscopy images that have unique global structures and observed improvement in the downstream segmentation accuracy when we used the generated images together with the raw images in the dataset. Our model may not offer any improvement over previously proposed parametric cell population simulation tools in cases where global structure is not observed (e.g., fluorescent nuclei images of cell cultures). Another limitation is that the proposed method is dependent on the pix2pix algorithm for generating the microscopy images from the masks. Even if we can almost perfectly simulate the distribution of the instances, the image-to-image translation task may not successfully generate such microscopy images in all cases.

We lastly acknowledge that we tested the performance of our method on a small number of images, which is a limitation to understanding the true generalizability of the accuracy and performance gains that we did observe. More extensive testing on larger datasets would be needed to paint a more comprehensive picture of model performance.

## STAR★Methods

### Key resources table

**Table:** 

REAGENT or RESOURCE	SOURCE	IDENTIFIER
**Deposited data**		

Main datasets (microscopy image and annotation) (salivary gland and fallopian tube)	Mund et al.	https://zenodo.org/record/8096773/files/datasets.zip
Main source code repository	This paper	https://doi.org/10.6084/m9.figshare.23791554
Synthesized data with SIMCEP and pix2pix	This paper	https://zenodo.org/record/8096773/files/mask_quality_experiment.zip
Pix2pix models (subset experiment, fold 0, 100%)	This paper	https://zenodo.org/record/8096773/files/pix2pix_models.zip
SIMCEP code	Lehmussola et al.^1^	https://zenodo.org/record/8096773/files/simcep.zip
StyleGAN2-ada trained models	This paper	https://zenodo.org/record/8096773/files/StyleGAN2-ada_models.zip
Synthesized training sets for the subset experiments (fold 0)	This paper	https://zenodo.org/record/8096773/files/subset_experiments_training_set.zip
Supporting data for the fine-tuning and the subset experiments	This paper	https://zenodo.org/record/8096773/files/Supporting_data_for_experiments_1_and_2.xlsx

**Software and algorithms**		

StyleGAN2-ada	Karras et al.^16^	https://github.com/NVlabs/stylegan2-ada-pytorch
pix2pix	Isola et al.^18^	https://github.com/junyanz/pytorch-CycleGAN-and-pix2pix
SIMCEP	Lehmussola et al.^1^	http://www.cs.tut.fi/sgn/csb/simcep/
StarDist	Schmidt. et al.	https://github.com/stardist/stardist
Cellpose	Stringer et al.^17^	https://github.com/MouseLand/cellpose
MATLAB R2020b (to run SIMCEP)	MathWorks, Inc	https://www.mathworks.com/products/matlab.html
Python 3.8.10	Python Software Foundation	https://www.python.org/

**Other**		

DSB 2018 dataset	Caicedo et al.^20^	https://bbbc.broadinstitute.org/BBBC038

### Resource availability

#### Lead contact

Further information and requests for the details of our method, please contact Dr. Peter Horvath (horvath.peter@brc.hu).

#### Materials availability

This study did not generate unique reagents.

### Experimental model and subject details

We used two single-cell datasets for testing the proposed method (Figure 1). One is a salivary gland tumor (acinic cell carcinoma) dataset referred to as salivary gland extracted from a 29-year-old male, healthy condition after 4 years of sample collection. No sign of mitosis, necrosis de-differentiation or perineural or intravascular growth are observed. The nuclei and a tumor marker IHC stained.^21^ The other is a fluorescently stained fallopian tube tissue extracted from a 64-year-old female (membrane and nuclei staining) named fallopian.^21^ The sample appears microscopically and histologically normal. In both datasets, the objects follow a specific layout that could be hard to explicitly generate using classic algorithms but using our approach, we can generate samples that are closer to the training dataset. (Figure 5) We also show qualitatively that using the StyleGAN2-ada generator, we are able to draw synthetic samples from this distribution, without any explicit parameterization or algorithm. (Figure 4)

#### Salivary gland tumor dataset (salivary gland)

The dataset consists of 10 annotated 3-channel images with resolution of 600x800 and contains a total of 1058 labeled cells. 5 + 5-folds are formed (5 for each experiment), each fold contains 8 images for training and 1 + 1 images for validation and testing. (Figure 1, top)

#### Fallopian tube dataset (fallopian)

This dataset originally consisted of 8 images and 1818 annotated cells split into 30 parts with varying sizes. Only the parts reaching the resolution 256x256 pixels are kept, therefore we finally got 17 images. The number of cells is counted in each part and are distributed to 8 groups to each contain roughly equal numbers of annotated cells. From the 8 groups, 4 + 4-folds are formed, each fold contains 6 groups for training and 1 + 1 groups for validation and testing. (Figure 1, bottom)

### Method details

#### Mask learning using GANs

##### Generative adversarial networks (GANs)

In traditional GANs^22^ the goal is to learn the mapping G:z→y where z is the element of the distribution of the training set. The learning employs two networks, the generator (G) and the discriminator (D), where both networks simultaneously improve to perform better in generating images (G) that cannot be distinguished from the real images by the discriminator, and the discriminator trained to do that task more successfully. Thus, the value function is V(G,D)=logD(y)+log(1−D(G(z)). V is minimized with respect to D and maximized with respect to G by doing one gradient update in each step. After the training has converged, G is used to generate samples from the distribution of the dataset and D is discarded. In the StyleGAN and its variants, the architecture of G is modified such that it progressively upsamples the image being generated while adds details to it. In each upsampling step, a style vector is used which is constructed by a multi-layered fully connected network from z. We chose the StyleGAN2-ada model to synthesize samples as it offers non-leaking augmentation that makes possible to train on datasets with limited size.

To train the StyleGAN2-ada model ($m_{StyleGAN2-ada}$), we used 256x256 pixel sized overlapping tiles extracted from each dataset. Only tiles containing at least 3 objects were kept. We used default settings (with augmentation turned on) when training the models. The actual parameters can be found in the deposited models.

The best model is selected based on Fréchet Inception Distance (fID) computed based on the pretrained ImageNet weights.^23^ Although ImageNet is a natural image dataset, it is shown that the features extracted by the model on medical images are also meaningful to assess the generated image quality.^16^ We observed that models with fID <100 produce synthetic masks that are numerically correct (they can be reconstructed by following the gradients without any significant error in the reconstructed mask). As the training progresses, the fID score may decrease but the variability of the objects may become less diverse, therefore visual assessment may also be needed.

In the case of the salivary gland dataset, the model used is trained from scratch, while the fallopian tube model is also tested by fine-tuning the salivary gland model for saving time and computational costs and also to demonstrate transfer capability of StyleGAN2-ada on the datasets.

##### Training from scratch

When training from scratch, the StyleGAN2-ada reaches the fID 54.33 at step 6500 on the salivary gland dataset, while the model converges on the fallopian tube after 12140 steps and reaches the lowest fID 56.45 (See Figures S1 and S2 in the supplemental information).

##### Transfer learning

When we used the checkpoint at step 5000 (5 million crops passed through the network) from the salivary gland model, we could fine-tune the network on the fallopian tube, and the network needs only 1800 steps (compared to the 12140 when trained from scratch) to reach the minimum score 46.61 that is also substantially better (17.4% lower distance from the ground truth compared to uninitialized training).

##### Fine-tuning with limited subsets

We used the salivary gland model at checkpoint 6400 (the best model on salivary gland) to fine-tune on the crops extracted from a limited number of training images from the fallopian tube dataset. We observed that the model has a good enough score even when less than 50% of the training set is used. See the details in supplemental information II: Table S2 and S3, the key resources table for the shared models and Table S4 in supplemental information for the descriptions of the shared models.

The StyleGAN2-ada is trained on the discretized flows that are 3 channel images thus no modification is needed in the code. The first channel encodes the object probability score (values 0 and 255 in the ground truth images). The gradient dx and dy (second and third channels) are discretized from [-1.0, 1.0] to the interval [0, 255].

#### Synthesizing the corresponding microscopy images

##### Image to image translation

The pix2pix method solves the image-to-image translation task using modified conditional GANs. Compared to a regular conditional GAN, the pix2pix method adds the dependency on the condition not only to the generator but also the discriminator. Thus, the value function becomes V(G,D)=logD(x,y)+log(1−D(x,G(x,z)), where the x is the condition (the source image), y is the target image and z is random latent vector. The loss function minimizes D and maximizes G by doing one gradient update for each input for both networks in each step. Once the training converges, the generator represents the mapping (x,z)→y.

##### Pix2pix training

we trained the models for 600 epochs on the salivary gland dataset and for 300 epochs on the fallopian tube. We used the model from the last epoch when synthesizing the samples (did not use validation set). All other parameters hold their default value. See the key resources table for the exact implementation we used.

StarDist instance segmentation: the masks in the training set are processed, and each object in the mask is converted into a star-convex polygon by first selecting the centroid of an object and then measuring the length of the rays connecting the contour of an object and its centroid. The angle between adjacent rays is equal and fixed for the entire dataset. The network consists only of convolutional layers (“U-Net” and "ResNet” are proposed), where the layer exactly before the top one branches to predict the probability map and the distance map. Both the probability and distance feature maps have spatial size proportional to the input size (or equal size if downsampling is not used). The probability map contains the object probability scores for each representative location, and the distance map at the same location represents the ray lengths encoding a candidate object (that is defined for the entire map). The training uses cross entropy loss to supervise the probability scores while uses mean squared error for the distances. During prediction, non-maximum suppression is used to find the best candidates.

##### StarDist training details

we set the batch size to 4, the number of rays to approximate the objects was 32 and the learning rate was 0.003. In the transfer learning experiment, we trained the models for 100 epochs. During fine-tuning we limited the training for 20 epochs, as the models usually converged after only a few epochs. We used the last model from the pretraining as the initial weights in the transfer learning experiment. We used the same parameters for the subset experiment except that all models were trained for 50 epochs.

Cellpose is an instance segmentation method that uses the heat-flow representation to encode each object in a labeled mask to a different image containing 3 channels: the probability map and a vector field encoding the flow. Since the heat-flow can be converted back to a labeled mask, the instance segmentation problem can be solved as a dense prediction task: the method uses a fully convolutional architecture (a “U-Net”-like network is used) to predict the flows that are converted back into labels.

##### Cellpose training details

we set the batch size to 8. In the transfer learning experiment, we trained the models for 100 epochs (during fine-tuning, we loaded the last model and trained for 20 epochs). In the subset experiment we disabled the rescaling based on the median object size and trained the models for 50 epochs.

##### Augmentation protocol

We used augmentations affecting only the input image and geometric transformations that affect both the input images and the corresponding masks. We apply random joint intensity change with coefficient sampled from uniform distribution U(0.6,2) and added bias sampled from U(−0.2,2) and apply standard Gaussian noise with strength 0.02. We observed that using this augmentation protocol alone degrades the performance in most of the cases. We apply random rotations and flips in each dimension with probability 0.5 independently. We also use elastic deformations applied in the original U-Net paper.^24^^,^^25^ The augmentations are applied on the fly during the training. We do not use the elastic deformations on the Cellpose masks as the flows for the deformated masks should have been computed before each step that is computationally too expensive (Table 1). We measure the generalization capability of our approach by also comparing it to basic augmentation pipelines.

##### Evaluation metric

We used a standard nuclei segmentation metric to evaluate the performance of our model.^1^ The metric matches the predicted and ground truth objects and computes their intersection over union (IoU). Then the size of true positives, false positives and false negative sets are computed on each IoU threshold from 0.5 to 0.9 with step size 0.05. The metric then computes the mean accuracy (TP over TP + FP + FN) over the thresholds.

### Quantification and statistical analysis

#### Synthesized mask quality quantification

The mask quality experiment is quantitatively evaluated by computing the fID for dataset pairs. We used the StyleGAN2-ada code for computing the fID initialized with ImageNet weights (see the key resources table for the StyleGAN2-ada availability). We did not directly compare the decoded masks but compared them in the heat-flow space.

#### Downstream task

We did cross validation for the downstream task experiments. In the subset experiments, we formed different subsets by removing items from each fold. In the subset experiments, each training is executed 10 times (training = one execution of a particular subset in a particular fold). In the transfer learning experiment, each training is executed 3 times. The accuracy of each single execution is deposited (see the resource availability and key resources table for details). For making the table in the fine-tuning and subset experiments, the trainings are averaged first on fold level, then the mean accuracy of the folds are reported. In each run, the model with the best validation score is considered for evaluation on the test set. The instance segmentation accuracy is computed using the StarDist metric implementation (see the key resources table). The standard deviation is also computed in the supporting data for the experiments (deposited, see the key resources table for details).

## Data Availability

• This study analyzes existing, publicly available data. All of the processed datasets reported in this paper are freely available using the links listed in the key resources table. • All original code reported in this paper is freely available via the links listed in the key resources table. • Any additional information required to reanalyze the data reported in this paper is available from the lead contact upon request.

## Supplemental information

Document S1. Tables S1–S5 and Figures S1–S3

Document S2. Article plus supplemental information
