# Supplementary material for: Structure preserving adversarial generation of labeled training samples for single-cell segmentation
Source: Cell Rep Methods. 2023 Sep 18;3(9):100592. doi: 10.1016/j.crmeth.2023.100592 (PMC10545934; doi:10.1016/j.crmeth.2023.100592)
Supplement: Document S1. Tables S1–S5 and Figures S1–S3 [file mmc1.pdf]

Cell Reports Methods, Volume *3*

Supplemental information

Structure preserving adversarial generation of labeled training samples for single-cell segmentation

Ervin Tasnadi, Alex Sliz-Nagy, and Peter Horvath

I. Related works

Reference	domain	task	uses unlabeled samples	evaluation metric
[1]	Medical images	Segmentation	No	DSC
[2,3]	Medical images	Segmentation	Yes	DSC
[4–7]	Medical images	Classification	No	Classification accuracy
[8]	Microscopy	Instance segmentation	Yes	Dice coefficient
[9]	Microscopy	Instance segmentation	Yes	AJI
[10]	Microscopy	Instance segmentation	Yes	mAP
[11]	Microscopy	Semantic segmentation	No	IoU
[12]	Microscopy	Instance segmentation	Yes	mAP
**Proposed**	**Microscopy**	**Instance segmentation**	**No**	**mAP**

Table S1. summary of existing GAN based augmentation methods related to Figure 2.

DSC stands for Dice Similarity Coefficient, AJI is the Average Jaccard Index, IoU is the pixel level Intersection Over Union, while mAP is the mean Average Precision (similar to the DSB 2018 metric). The table is a summary of the introduction section in the main text.

II. StyleGAN2-ada training details

Training from scratch:

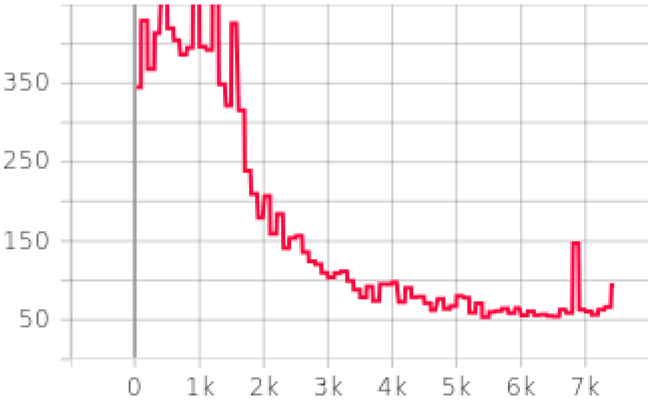

Figure S1: Progression of the fID score when training the `salivary gland` StyleGAN2-ada model trained from scratch related to STAR methods.
The horizontal axis is the number of images already shown to the model.

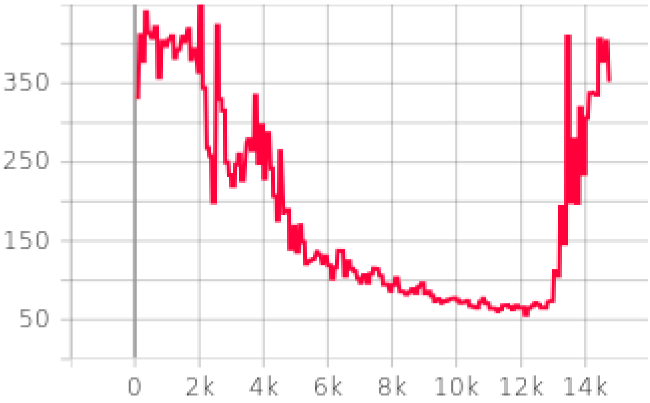

Figure S2: Progresson of the fID score when training the `fallopian tube` StyleGAN2-ada model trained from scratch related to STAR methods.
The horizontal axis is the number of images already shown to the model.

	**step**	**fID**
fallopian tube	12140	65.45
salivary gland	6500	54.33

Table S2: Minimal fIDs when StyleGAN2-ada is trained from scratch on the datasets related to STAR methods.
One step means that 1000 crops are passed through the network. The fID is the minimum fID reached during the training.

Fine-tuning

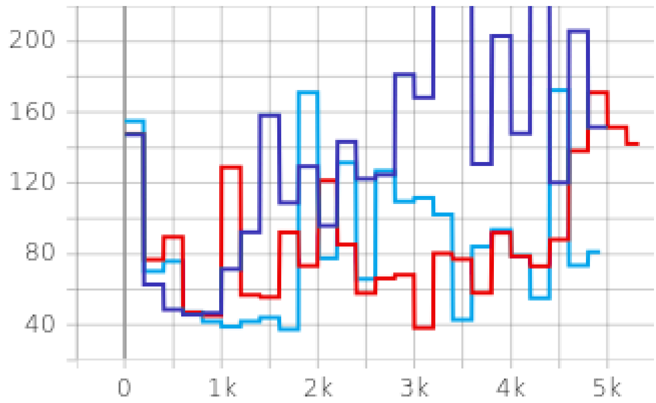

Figure S3: StyleGAN2-ada fine-tuning experiments: salivary gland → fallopian tube related to STAR methods.
Cyan: subset size=13; red: subset size=9; dark blue: subset size=6. The plot shows the fID score as a function of the number of images already shown to the model.

subset size	fID @ step 1000	min fID
6	46.74	37,43
9	45.36	38,34
13	41.86	45,79

Table S3: fID scores during fine-tuning a `salivary gland` model on the `fallopian tube` dataset related to STAR methods.
The salivary gland model is captured at step 5000 when trained on fold 0. The fID score does not degrade too much even if we use less than 50% of the samples for fine tuning. The model produces images with a reasonably good fID after only 1000 steps.

The summary of the deposited models:

model id	dataset	description	step	fID (@step)	best fID
`00014-masks_cp-auto2-kimg25000-ada-target0.6-bg`	fallopian tube	trained from scratch, best model	@12,14k	56,45	56,45
`00000-salivary_dry_tiled_aug_256_cp-auto2-kimg25000-ada-target0.6-bg`	salivary gland	trained from scratch, best model	@6,5k	54,33	54,33
`00000-masks_cp-auto1-kimg25000-ada-target0.6-bg-resumecustom`	fallopian tube	trained from salivary-5000, best model	@1,8k	46,61	46,61

00004-13-auto1-kimg25000-ada-target0.6-bg-resumecustom	fallopian tube	trained from salivary-6400; fold 0, lim 13, 1k step	@1k	41,86	37,43
00003-9-auto1-kimg25000-ada-target0.6-bg-resumecustom	fallopian tube	trained from salivary-6400; fold 0, lim 9, 1k step	@1k	45,36	38,34
00002-6-auto1-kimg25000-ada-target0.6-bg-resumecustom	fallopian tube	trained from salivary-6400; fold 0, lim 6, 1k step	@1k	46,74	45,79

Table S4: The summary of the deposited models related to STAR methods.

III. Subset experiments quantitative results

See the resource availability section of the main article for the supporting data.

		Fallopian tube / subsets				Salivary gland / subsets		
network	synthetic	3	6	9	13	3	5	8
Cellpose	+	0.2754 (**+0.0434**)	0.2906 (**+0.062**)	0.3132 (**+0.0247**)	0.3122 (**+0.093**)	0.3866 (**+0.1261**)	0.4215 (**+0.1596**)	0.4358 (**+0.1062**)
Cellpose	-	0.2320	0.2844	0.2885	0.3029	0.2605	0.2619	0.3295
StarDist	+	0.2837 (**+0.0930**)	0.3056 (**0.0415**)	0.3154 (**0.0256**)	32.30 (**0.0162**)	0.2867 (**+0.038**)	0.3021 (**+0.0378**)	0.3079 (**+0.0291**)
StarDist	-	0.1906	0.2641	0.2898	0.3068	0.2487	0.2643	0.2788

Table S5: The numbers are the mean of the accuracies computed on each fold related to STAR methods and figure 6.
The baseline numbers for the Cellpose are significantly lower compared to Experiment 1, since we disabled the input uniformization for a fairer comparison with StarDist. Green numbers: improvement by our method. The raw numbers are in the file "Supporting_data_for_experiments_1_and_2.xlsx" in the attached repository.

References

1. Bowles, C., Chen, L., Guerrero, R., Bentley, P., Gunn, R., Hammers, A., Dickie, D.A., Hernández, M.V., Wardlaw, J., and Rueckert, D. (2018). Gan augmentation: Augmenting training data using generative adversarial networks. arXiv preprint arXiv:1810.10863.

2. Chaitanya, K., Karani, N., Baumgartner, C.F., Becker, A., Donati, O., and Konukoglu, E. (2019). Semi-supervised and task-driven data augmentation. In Information Processing in Medical Imaging: 26th International Conference, IPMI 2019, Hong Kong, China, June 2–7, 2019, Proceedings 26 (Springer), pp. 29–41.

3. Sandfort, V., Yan, K., Pickhardt, P.J., and Summers, R.M. (2019). Data augmentation using generative adversarial networks (CycleGAN) to improve generalizability in CT segmentation tasks. Scientific reports *9*, 16884.

4. Frid-Adar, M., Diamant, I., Klang, E., Amitai, M., Goldberger, J., and Greenspan, H. (2018). GAN-based synthetic medical image augmentation for increased CNN performance in liver lesion classification. Neurocomputing *321*, 321–331.

5. Sundaram, S., and Hulkund, N. (2021). Gan-based data augmentation for chest x-ray classification. arXiv preprint arXiv:2107.02970.

6. Frid-Adar, M., Klang, E., Amitai, M., Goldberger, J., and Greenspan, H. (2018). Synthetic data augmentation using GAN for improved liver lesion classification. In 2018 IEEE 15th international symposium on biomedical imaging (ISBI 2018) (IEEE), pp. 289–293.

7. Madani, A., Moradi, M., Karargyris, A., and Syeda-Mahmood, T. (2018). Chest x-ray generation and data augmentation for cardiovascular abnormality classification. In Medical imaging 2018: Image processing (SPIE), pp. 415–420.

8. Majurski, M., Manescu, P., Padi, S., Schaub, N., Hotaling, N., Simon Jr, C., and Bajcsy, P. (2019). Cell image segmentation using generative adversarial networks, transfer learning, and augmentations. In Proceedings of the IEEE/CVF conference on computer vision and pattern recognition workshops, pp. 0–0.

9. Mahmood, F., Borders, D., Chen, R.J., McKay, G.N., Salimian, K.J., Baras, A., and Durr, N.J. (2019). Deep adversarial training for multi-organ nuclei segmentation in histopathology images. IEEE transactions on medical imaging *39*, 3257–3267.

10. Hollandi, R., Szkalisity, A., Toth, T., Tasnadi, E., Molnar, C., Mathe, B., Grexa, I., Molnar, J., Balind, A., Gorbe, M., et al. (2020). nucleAIzer: a parameter-free deep learning framework for nucleus segmentation using image style transfer. Cell Systems *10*, 453–458.

11. Pandey, S., Singh, P.R., and Tian, J. (2020). An image augmentation approach using two-stage generative adversarial network for nuclei image segmentation. Biomedical Signal Processing and Control *57*, 101782.

12. Liu, Y., Wagner, S.J., and Peng, T. (2022). Multi-modality microscopy image style augmentation for nuclei segmentation. Journal of Imaging *8*, 71.
